# Assessment of the onset of analgesia and length of analgesia following the use of PBM with different wavelengths: a clinical study

**DOI:** 10.1007/s10103-024-04184-y

**Published:** 2024-09-19

**Authors:** Sachin Kulkarni, Laurence J. Walsh, Yash Bhurani, Roy George

**Affiliations:** 1 School of Medicine and Dentistry, Griffith University, Gold Coast, Queensland Australia; 2 School of Dentistry, University of Queensland, Brisbane, Queensland Australia; 3 School of Dentistry, University of Adelaide, Adelaide, South Australia Australia

**Keywords:** Photobiomodulation, Infrared, Analgesia, LED, Dental pulp, Wavelength

## Abstract

This clinical study assessed photobiomodulation (PBM) induced analgesic effects of diode lasers and an LED light source on the dental pulp. Baseline responses to electric pulp testing (EPT) were recorded in 93 healthy unrestored premolar teeth in 26 adults (age range 22–63 years) attending a private dental practice. The teeth were irradiated on buccal and lingual aspects of the crown, by placing the tips on the middle third of the crown of the teeth, on separate days for each of 4 different light sources (660, 808, or 904 nm diode lasers, or a novel multi-wavelength LED light source (700–1100 nm)) using comparable parameters (100 mW, 30 s, 6 J). EPT scores were measured after a further 1-, 2-, 5- and 20-min. Discomfort caused by PBM therapy was recorded using the Wong-Baker scale. EPT changes were tracked over time using repeated measures analysis of variance. Baseline EPT scores were very consistent between different days (linear regression r^2^ 0.9422–0.9648). All PBM devices caused a significant elevation in EPT at 5 min, with an earlier onset at 2 mins for 904 nm and LED. The LED was the only light source that elevated scores at 20 min. Across 2–20 min, when ranked by effectiveness, the greatest EPT elevations were seen for LED, followed by 904 nm, then 660 nm and finally 808 nm. Discomfort during PBM was most common with 904 nm, followed by 808 nm. No discomfort occurred from the LED. Among the light sources utilized, the LED multi-wavelength system demonstrated the largest increase in EPT readings, suggesting its potential as a non-pharmacological alternative for achieving dental analgesia compared to diode lasers.

## Introduction

Analgesia refers to the phenomenon of blocking afferent nerve impulses for pain, while leaving other sensations intact. Pain impulses can be triggered from injury and by noxious stimuli acting on nociceptors [1]. The dental pulp is well endowed with nociceptors, and dental pulp pain (toothache) is a common and major source of morbidity [2]. The dental pulp contains primary nociceptive fast conducting myelinated A-δ fibres and slow conducting unmyelinated C-fibres, with the more plentiful A-δ fibres located primarily at the pulp-dentine border in the coronal portion of the pulp, and especially in the pulp horns [3]. The free endings of these nerves are highly responsive to cold and to hypertonic conditions [4], giving a well localised short, sharp pain [2]. Conversely, the C fibres respond to heat with a poorly localised dull, lingering pain. The remaining A-beta fibres respond to vibrations. Hence, for dental procedures that may cause discomfort, an analgesic action based on blocking transmission of A-δ fibres would be desirable, to enhance patient comfort [5, 6].

Photobiomodulation (PBM) can cause analgesia by blocking the conduction of action potentials along sensory nerve fibres through effects on ion channels, but without causing injury to nerves. This effect has been demonstrated conclusively in cultured neurones [7]. Another proposed potential mechanism for PBM-analgesia involves transient temperature elevations blocking ion channel function [8], as a photothermal process (Hodgin Huxley mechanism), although this concept remains controversial [9]. Away from the direct site of irradiation, beta-endorphin release and depressed release of arachidonic acid molecules can also occur, albeit on a longer time scale [10].

PBM for the dental pulp requires efficient transmission through dental hard tissues, to reach the nerves and nerve endings in the dental pulp. Dentine sclerosis with age and the downstream optical changes in the dentine from anisotropic to isotropic mean that the extent of sclerosis can affect transmission greatly, with loss through scattering caused by crystals of hydroxyapatite mineral [11]. As well, dentine has a heterogeneous structure, with larger fluid filled tubules closer to the dental pulp [12]. Overall, transmission of light through dentine and enamel is greatest in the near infrared range and improves as one leaves the visible red region and enters the near infrared region [11–15]. This “transmission window” is similar to that which occurs in skin and soft tissues, since absorption of light by water is very low in the visible red and near infrared regions [14]. From these considerations, PBM devices that emit light at the edge of the visible spectrum (700 nm) or in the near infrared (700–1400 nm) are candidates for PBM for the dental pulp.

A recent systematic review identified the need for further work to optimize PBM analgesia for dental applications in terms of the light parameters, including the wavelength [16]. Hence, the aim of this clinical study was to assess, for the first time, analgesic actions in the dental pulp in healthy unrestored teeth, using 3 different diode lasers and a novel LED device that emits light across part of the infrared region.

## Materials and methods

This clinical trial was designed in line with Therapeutic Goods Administration (TGA) annotated International Council for Harmonisation of Technical Requirements for Pharmaceuticals for Human Use (ICH) Guideline for Good Clinical Practice [17]. The study was approved by the Griffith University Human Research Ethics Committee (Ref 2022/668) and was registered with Australia New Zealand Clinical Trial Registry (ACTRN 12623001143617).

### Participant selection

The study was designed on the basis that electric pulp testing (EPT) values are stable in the same individual tooth over periods of months. A power analysis was undertaken (StatMate, GraphPad Software, San Diego, CA, USA) using a correlation r^2^ value of 0.94 and a standard deviation of 15, which were obtained from a pilot study with 10 subjects. On the basis that each subject would have at least one pair of premolars, a sample size of 20 subjects would give an 80% power to detect a difference between means of 3.35, with a significance level (alpha) of 0.05 (two-tailed). A total of 26 subjects were recruited, to allow for potential dropouts.

To eliminate confounding effects of prior restorations, the selection criteria for the study were designed to include healthy adult subjects (aged at least 18 years) with paired unrestored healthy premolar teeth, using selective convenience sampling from a private dental practice. The teeth were required to have no past history of trauma, restorations, or other dental treatment. Patients were required not to be taking medications that could modulate pain. Exclusions included pregnancy, mental illness, neurological conditions, oral mucosal disease, lack of capacity to give consent, cognitive impairment, and intellectual disability.

### Workflow

At the first appointment, after obtaining informed consent, the shade of the teeth to be treated was measured (VITA Easyshade^®^ Compact, VITA Zahnfabrik H. Rauter GmbH & Co., Bad Säckingen, Germany). A practice run for EPT was conducted using a tooth not involved in the study, to familiarise the participant with the EPT procedure and the accompanying sensations. FLAIRESSE Prophy Fluoride Gel^®^ (DMG, Bielefeld, Germany) was used as a conducting medium. The baseline EPT value was then recorded for the teeth to be treated. Past studies were used as the basis for the EPT methodology [18] however for this study the EPT system (Digitest^®^ 3, Parkell, Edgewood, NY, USA) had a wider range of up to 500 volts recorded at the highest possible digital reading of 64. The Analytic EPT (Analytic Endodontics, Sybron Dental Specialties Inc., Orange, CA, USA) used in previous studies had range of only 300 volts recorded at highest possible digital reading of 80. The Digitest^®^ 3 in this study was set to a peak power of 250 µA and a pulse duration of 60.5ms to ensure that patients could quickly and distinctly perceive the onset of the EPT stimulus. This was evident from the consistently similarly three readings when teeth were assessed for their baseline responses. Participants were instructed to release their hand off the lip clip of the EPT testing unit immediately they perceived a mild or tingling sensation in the tooth. Doing this broke the current flow and stops the measurement process, at which point the value was recorded. EPT testing was repeated three time at the baseline to assess the accuracy of the testing protocol, with a gap of at least 4 min between testing runs. The workflow then involved treating each included tooth with one of the four PBM light sources, and then recording EPT scores at 1, 2, 5 and 20 min after PBM. As well, subjects recorded their level of discomfort during PBM using the Wong Baker faces pain rating scale [19].

This workflow was repeated at later visits, treating the same teeth in the same locations, and using the same manner on different days, but with different PBM devices, and on each day, measuring the EPT scores at baseline and then over the 20 min period after irradiation.

### PBM devices

A total of four PBM light sources were used – three semiconductor diode lasers (660 nm (Klas-D 660, Konf^®^, Konftex, Taiwan), 808 nm (Klas-D 660, Konf^®^, Konftex, Taiwan), and 904 nm (Mid-Laser, Irradia^®^, Sweden) and one multi-wavelength LED light source (Nuralyte^®^, Dentroid, Canberra, Australia) (Table 1). As shown by testing using a spectrometer (USB 2000, Ocean Optics, Dunedin, FL, USA), the LED device generated light from 700 to 1100 nm, with emission peaks at 770, 850 and 950 nm. The PBM devices had flat top handpieces, and they were placed in contact mode onto the middle third of the tooth surface, covering the crown of the tooth on both buccal and lingual/palatal aspect. The device output was measured in between every tooth that was irradiated.

**Table 1:** PBM light source specifications

Parameters	Konf^®^ Klas-D61	Konf^®^ Klas-D81	Irradia^®^	Nuralyte^®^ LED
Wavelength(s)	660 nm	808 nm	904 nm	700–1100 nm
Power	100 mW	110 mW	90 mW	110 mW
Measured output	81.8 mW	100.1 mW	32.9 mW	113.0 mW
Measured spot size and calculated area	6 mm 0.28 cm^2^	6 mm 0.28 cm^2^	6 mm 0.28 cm^2^	7 mm 0.39 cm^2^
Power density	289 mW/cm^2^	354 mW/cm^2^	116 mW/cm^2^	293 mW/cm^2^
Total energy (for 60 s)	4.91 J	6.01 J	1.97 J	6.77 J
Energy density (for 60 s)	17.4 J/cm^2^	21.3 J/cm^2^	7.0 J/cm^2^	17.6 J/cm^2^
Mode of operation	Continuous	Continuous	5000 Hz pulsed	Continuous

Details of the light sources are given in Table 1. Optical power emissions were assessed using a laser power meter (SpectraMet, Laserdyne Technologies, Molendinar, Australia). The intent of the settings used was to achieve an energy density of around 8–10 J/cm^2^ for each of the buccal and lingual exposure runs, and to have similar values for power density and energy density across all 4 light sources, so that the photon flux would be similar, whilst keeping the exposure periods within a clinically useful time of 30 s.

For each light source, the energy was delivered to the tooth crown, for 30 s from the buccal aspect, and then for a further 30 s from the lingual/palatal aspect. The round spot of light was positioned to cover the centre of the premolar tooth surface being treated, with the tip held against the tooth surface in contact mode. All units had similar curved delivery tips, allowing intra-oral delivery of light for PBM with the tip end placed directly against the enamel. The tip curvatures measured from the main axis of the handpiece were 60 degrees, except for the Irradia 904 nm, which was 45 degrees.

Risks of energy from PBM light sources to study participants and to the operator were assessed, evaluated, and mitigated or minimised, by using optical protection and by appropriate engineering and environmental controls, as per the relevant Australian national laser safety standard (AS 4173:2018 Safe use of lasers and intense light sources in health care).

### Data analysis

All analyses used Instat software version 3.1 (GraphPad, San Diego, CA, USA) with *p* < 0.05 as the threshold for significance. Data sets for continuous variables were assessed for normality using the Kolmogorov-Smirnov test. EPT scores for individual teeth were tracked from baseline to post-exposure time points, for each light source, using repeated measures analysis of variance, with comparisons made to baseline using post-hoc tests to assess the effect of time. Likewise, comparisons were made between different light sources for the same tooth using repeated measures analysis. Data for EPT scores were also expressed as the percent change from baseline and categorized in terms of the amount of increase. Categorical data for response patterns and for Wong Baker discomfort scores were analysed using the Chi square test.

To assess the reliability of the clinical model, baseline EPT scores were compared within an individual subject across 4 different days for the four different light sources (660 nm versus 808 nm, 904 nm, or LED), using least squares linear regression, and the coefficient of determination (r^2^) and gradient of the line of best fit calculated.

Response patterns for the same subject between different light sources were compared to identify non-responders. This followed the recommendations of the Initiative on Methods, Measurement, and Pain Assessment in Clinical Trials (IMMPACT) [20–22] The number needed to treat (NNT) was calculated, as this could be a clinically useful measure for clinicians to appreciate the amount by which PBM can influence pulp sensibility, as measured by EPT. The threshold used for this calculation was an elevation in EPT of 25% [23, 24].

## Results

### Subjects

A total of 26 subjects (12 males, 14 females; age range 22–64 years) were enrolled and completed the study. Most subjects (19/26) had 4 matched premolar teeth for analysis which met the selection criteria of being sound, unrestored, and free of problems such as dentinal hypersensitivity, while 4 subjects had 3 premolars, 2 had 2 premolars and one subject had a single premolar. This gave a total of 93 sites, each of which was used to test all 4 light sources, but on different days. All the included teeth in the study were within the normal tooth colour range of the Vita Shade guide.

**Fig. 1:**
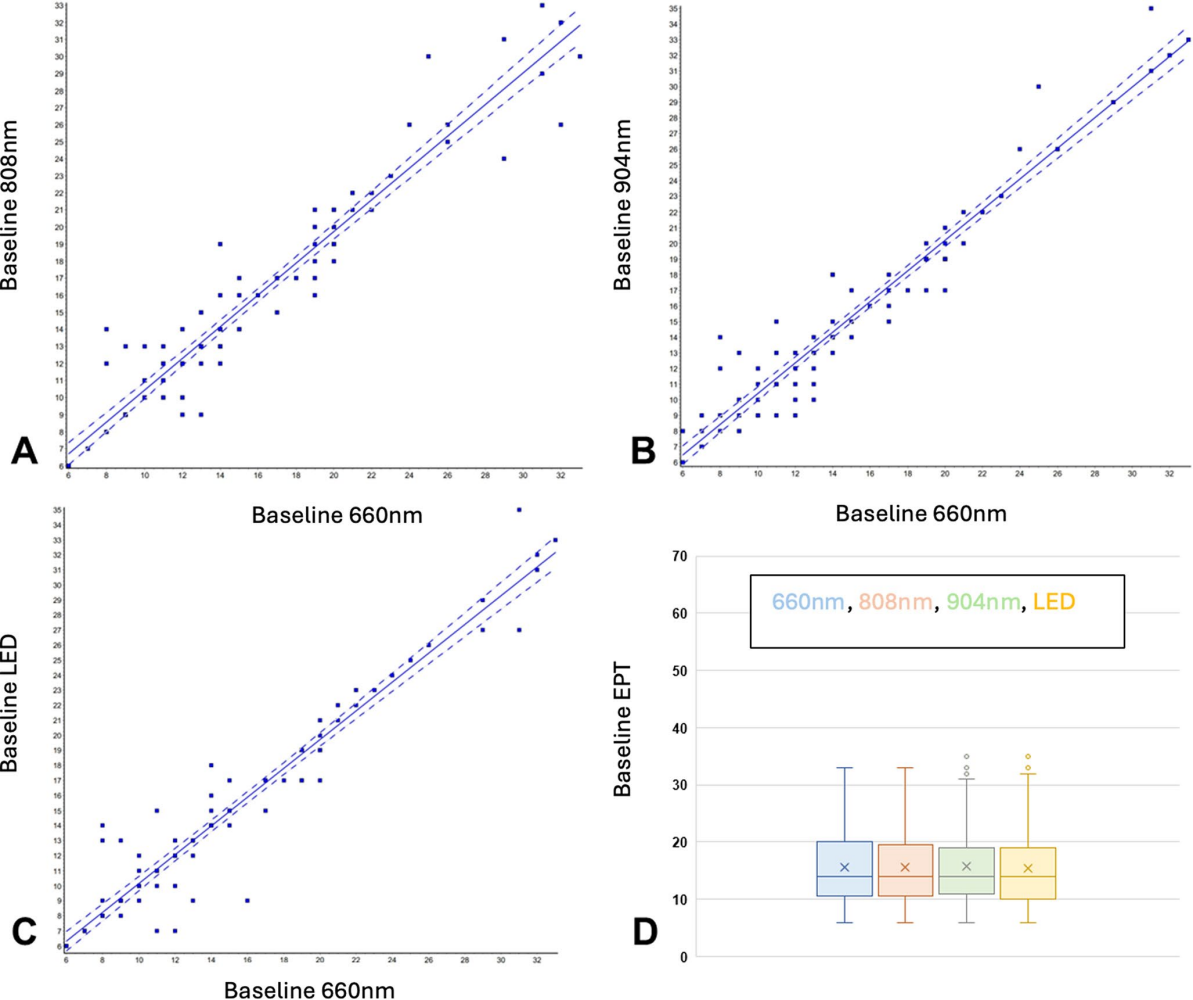
Reproducibility analyses for baseline EPT readings. Panels **A**-**C** Least squares linear regression analyses comparing baseline EPT scores recorded on teeth before 660 nm photobiomodulation (PBM) with baseline scores recorded before treatments with 808nm, 904nm and LED Label **D** is a box and whisker plot showing baseline scores across the 4 visits, prior to PBM treatment

When baseline EPT scores were compared within an individual subject across 4 different days for the four different light sources, strong consistency was noted, which indicated good standardisation of tooth responses and EPT techniques (*N* = 93 sites; Baseline 660 nm vs. 808 nm r^2^ = 0.92, slope = 0.93, Baseline 660 nm vs. 904 nm r^2^ = 0.94, slope = 0.98; Baseline 660 nm vs. LED r^2^ = 0.93, slope = 0.96) (Fig. 1). Using the Friedman Test (nonparametric repeated measures ANOVA, comparing the baseline measurements across 4 days the p value was 0.3300, and considered not significant.

### Action spectrum

**Fig. 2:**
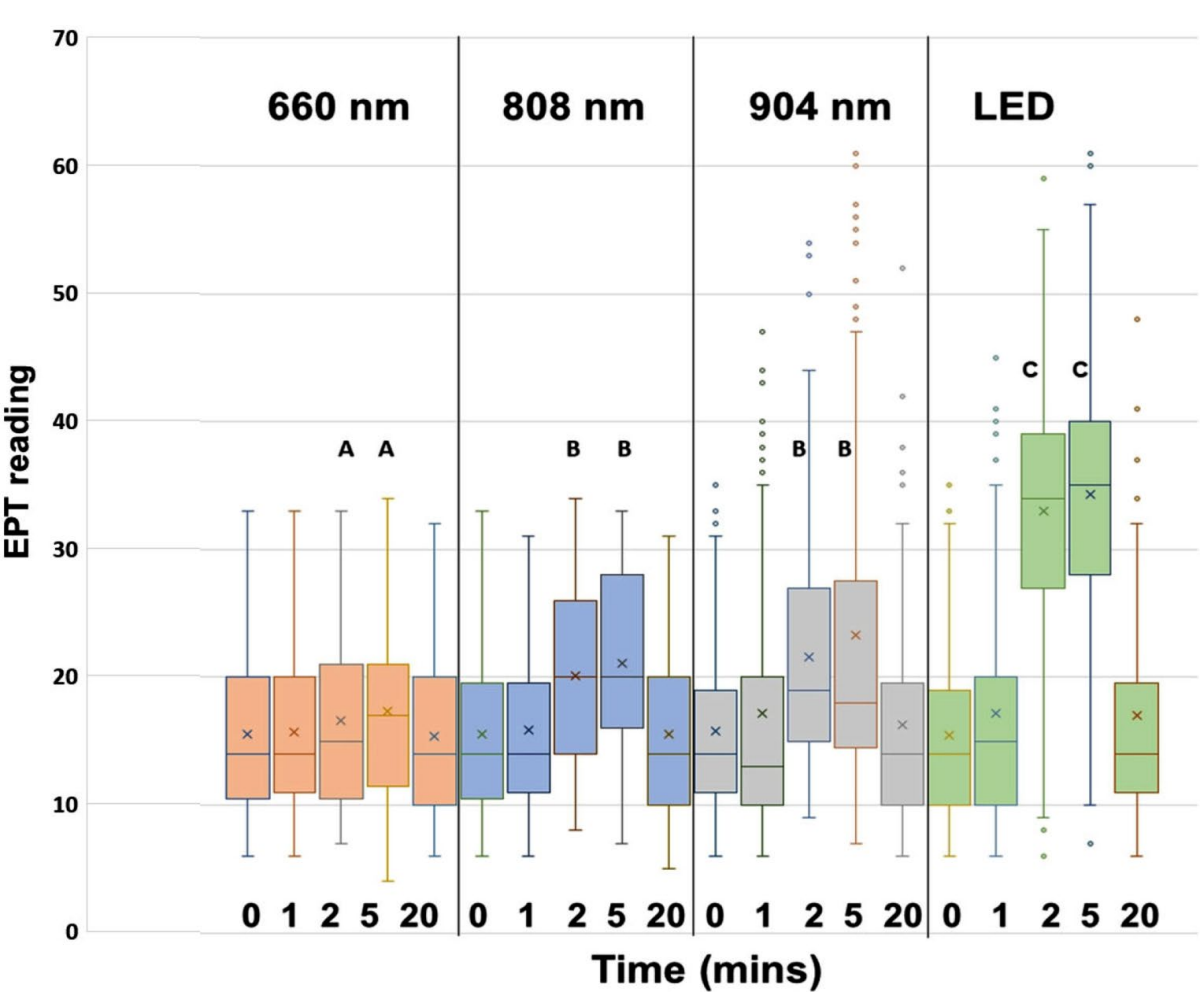
EPT scores over time in each of the PBM groups. Upper case letters show groups that were significantly above the baseline of time zero. Different letters denote significant differences between groups. In the box and whiskers plots, the box shows the first and third quartiles. The horizontal line is the median, while the X symbol represents the mean

When EPT scores were assessed across multiple time points for all 93 sites using nonparametric repeated measures ANOVA, near infrared PBM light sources elevated EPT thresholds over time (*p* < 0.001, Fig. 2). After reaching at peak at 2–5 min, EPT readings returned to baseline at 20 min. All 4 light sources gave a statistically significant elevation in EPT from baseline at 5 min (*P* < 0.05 using the Friedman test and Dunn’s Multiple Comparison Test).

**Fig. 3:**
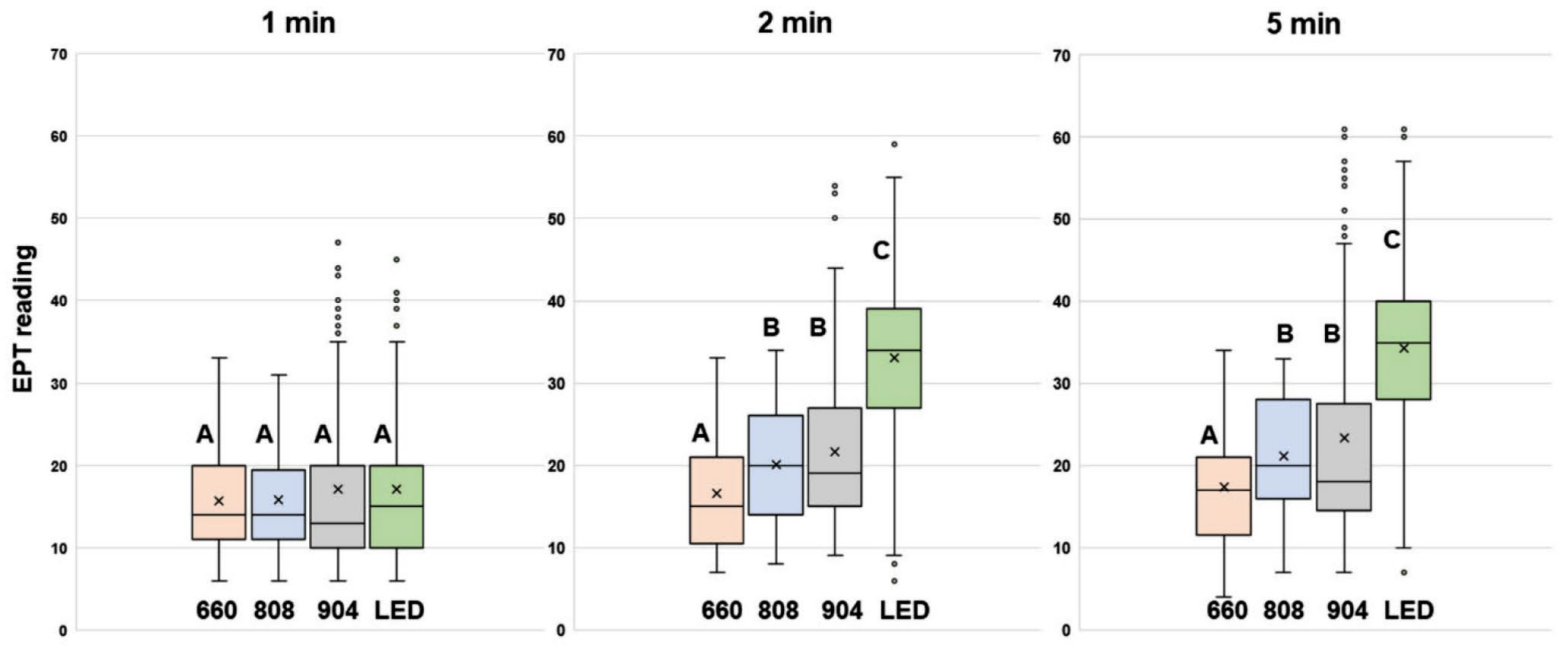
Onset of elevations in EPT scores with different PBM treatments. Upper case letters (**A**, **B** and **C**) denote significant differences from each other (A, B and C) between groups both at a given time point and across different time points

When comparing different PBM light sources, the elevation in EPT scores was greatest at 2 min and 5 min for LED (*p* < 0.001), followed by 904 and 808 nm, which were not significantly different from one another (Fig. 3). Differences between baseline and readings at 1 min and 20 min were not significant in any group.

Based on the time taken to perform EPT, it was estimated that the onset of the effect for the 904 nm laser and the LED was between 1 and 2 min following PBM, and 2–5 min for the 808 nm and 660 nm lasers (Fig. 3). The PBM-induced elevation in EPT scores fell away after its maximum at the 5-min time point, declining towards baseline. At the 20-minute mark, all readings for 4 light sources had declined to the point that differences between 20 min and baseline were not statistically significant, thus indicating a return of normal dental pulp sensibility.

### Responder analysis

At the subject level, all 26 subjects responded to PBM with elevated EPT readings. At the tooth level, only 1 of 93 teeth was unresponsive to all 4 light sources. Of note, a further 5 teeth were unresponsive to all 3 lasers, but each of these 5 teeth responded well to the LED device.

**Fig. 4:**
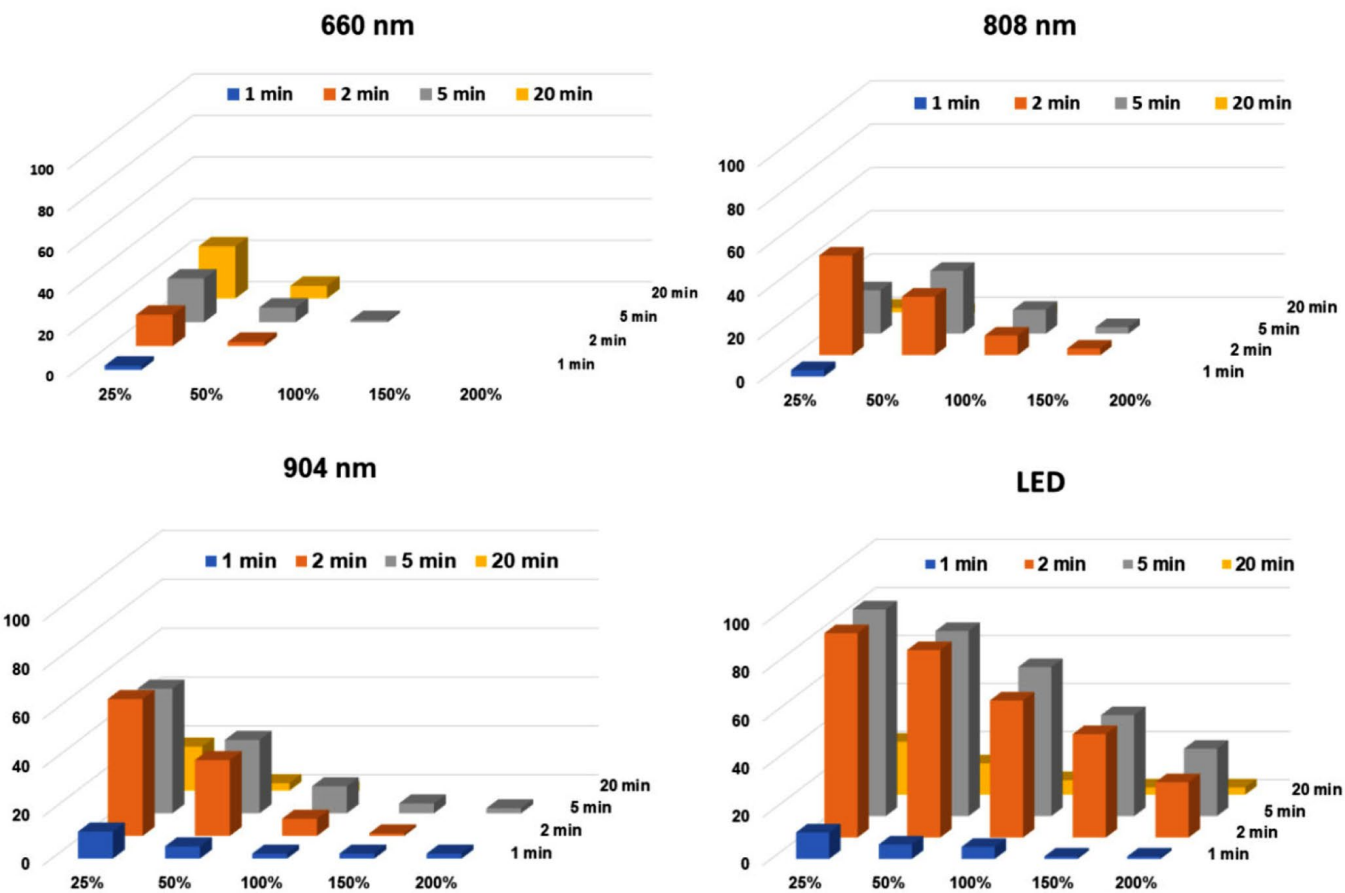
Responder patterns for the 4 different PBM devices, when responses were expressed as a proportion of increase over the baseline EPT value (at least 25%, at least 50%, and so on). The vertical scale is the frequency, which is the number of sites (out of 93) achieving at least the stated level of increase in EPT at the stated time points. Note the higher predictability of the LED compared to the diode lasers

When analysing data at the site level by the extent of increase in the EPT (measured as a percentage of the baseline EPT reading), the overall effectiveness was rated (from most to least) as LED > 904 nm > 808 nm > 660 nm, with differences between devices being significant at 2 min and at 5 min (*p* < 0.001, Fig. 4). Thus, analgesic actions improved with the use of longer wavelengths.

### NNT for superiority of the LED over diode lasers

When comparing the best performing diode laser (904 nm) and the LED, the number needed to treat with the LED was 8, to ensure a more 25% elevation in EPT over and above that achieved with the 904 nm laser.

### Discomfort analysis

The Wong-Baker faces pain scale was used to report discomfort experienced during the application of PBM. Of the 132 experimental runs, 29 caused discomfort, with every patient reporting discomfort in at least one tooth, except for the one subject that only had 1 tooth included in the study. There was a significant effect of light source on the prevalence of discomfort (*p* < 0.0001). No subjects reported discomfort for the LED light source. The most likely light source to cause discomfort was the 904 nm laser, followed by the 808 nm laser, and then the 600 nm laser. All but one subject who reported discomfort for 904 nm laser also reported discomfort for the 808 nm laser. An analysis of gender indicated that females were more likely to report discomfort than males (*p* = 0.018).

## Discussion

The results of the present clinical study expand the evidence base for PBM analgesic effects on the dental pulp by showing that, for healthy premolar teeth, elevated EPT readings can be gained by using LED light sources as well as diode lasers, with the effect being more pronounced when light sources with longer wavelengths are used. While both the LED device and the 904 nm laser caused the most predictable elevations in EPT, several key differences were noted, including the greater efficacy of the LED source, its faster onset, and its lack of discomfort, when compared to the 904 nm laser. This information can help inform decisions around the choice of light sources for pre-emptive analgesia, where irradiation precedes a procedure that is likely to cause discomfort.

A strength of the present study was the high reproducibility of the baseline EPT scores in individual teeth from day to day, and the use of paired contra-lateral teeth. This is important as it allows reliable comparisons of the effects of different PBM light sources across different days. There were no effects of tooth type (first or second premolar) or left versus right side on the analgesic actions for any light source. Despite this, it is important in future studies to assess whether the parameters for irradiation need to be adjusted for larger teeth, such as first molars. Likewise, it will be essential to assess how the presence of existing restorations in teeth influences their susceptibility to PBM analgesia, and likewise the responses for teeth that have existing low levels of inflammation, such as that caused by dental caries that has reached the dentine. This latter situation of having to deal with a tooth with past treatment or existing issues is common in clinical practice.

Another noteworthy consideration is the necessity for expanding the selected time intervals utilized in the present study in future investigations. This expansion would offer a more comprehensive understanding of the initiation and duration of photobiomodulation (PBM) analgesia across different light sources and irradiation parameters. It is proposed that this extension encompass time points both preceding the 1-min mark and extending beyond the 20-min interval. Such an approach would provide insights into the practical duration of PBM analgesia for dental pulp, potentially facilitating various straightforward dental procedures, such as tooth scaling and simple intra-coronal restorations within the estimated window of 15–20 min. Additionally, exploring protocols involving re-application of PBM to achieve subsequent periods of analgesia warrants further investigation through clinical studies.

In the present study, the intention was to use comparable exposure parameters for each light source that would achieve the desired energy density of 8–10 J/cm^2^ in a 30 s period. This was achieved for three of the four light sources, with the exception being the 904 nm laser, where the stated power setting of 90 mW resulted in only a measured average power of 33 mW being delivered from the tip. This could be because of lower production of laser energy from the source, losses in the delivery system, or, more unlikely, procedural errors in laser power measurement.

In this clinical trial, the stronger influence of longer near infrared wavelengths on EPT readings can be explained from greater penetration of these, and activation of different photoreceptors. The measured change in EPT readings inform the extent to which the various wavelengths of light influence the firing of neurones [25]. Light that is absorbed passing through tooth structure will cause more photothermal effects, leading to patient discomfort, and fewer photons will reach the dental pulp. One study has shown that light transmission through enamel and dentine is two times better for 980 nm than 650 nm [26], which explains the greater average effect and higher predictability of the longer near infrared light sources than the visible red 660 nm light source. Past studies have shown that 1064 nm light from the Nd: YAG solid state laser is suitable for elevating EPT readings and causing analgesia [27, 28]. Based on this, the action spectrum for PBM analgesia in the near infrared region extends to at least 1064 nm.

The present results for the LED unit indicate there is value in the concept of a PBM light source that emits more than one light wavelength. The concept of using two or more light wavelengths as a preferred way to achieve PBM analgesia compared to single wavelength lasers has appeared in previous literature [29]. This suggestion has arisen from the fact that visible red light (600–700 nm) is absorbed in superficial regions of soft tissue, while longer wavelengths (e.g. 780–950 nm) penetrate better and so can reach deeper targets within soft tissue [29]. The present results reinforce this concept, in the setting of dental hard tissues, though the superior performance of the LED system with multiple dominant wavelengths. Further work is needed to explore the value of this LED system in terms of PBM analgesia in soft tissues. As well, in that context, the influence of factors such as vascularity and tissue pigmentation or colour need to be considered as possible variables that will influence the PBM action.

In the present study, the shade of teeth within the normal range of the traditional Vita™ shade scale did not influence the outcomes for PBM analgesia. Separate studies are needed to assess light penetration in situations where teeth have intense discolouration, since that can be expected to increase attenuation by absorption, with consequences of greater warming (through photothermal actions), and correspondingly less PBM actions. In relation to dentine, while conventional dental radiographs can indicate if atrophy of the dental pulp has occurred with age, there are no current simple methods to quantify how much sclerosis has occurred within the dentine of a tooth. Sclerosis can occur due to increasing age, the placement of restorations, tooth grinding and clenching habits, or dental trauma [30]. Variations in the extent of sclerosis could explain why within the one patient, premolar teeth of similar size did not have exactly the same EPT readings at baseline, nor did they always show the same magnitude of response to a particular PBM light source. Importantly variations in EPT devices with their different peak power and pulse duration can make comparison between different studies difficult. Past work also has shown that the transmission of near infrared light varies by wavelength in dentine [31]. Further analysis of the effect of sclerosis on PBM analgesia should be undertaken.

Finally, future work should also consider situations where the tooth being treated has sensitive cervical dentine, such that caused by non-carious tooth structure loss from acid erosion, or by abrasion by toothbrushing. It is noteworthy that the inclusion criteria applied in the current study excluded teeth exhibiting sensitivity or dental caries. It is plausible that the response patterns of dental pulp to photobiomodulation (PBM) analgesia may vary for teeth presenting with sensitivity, whether attributable to exposed cervical dentine or dental caries, compared to those deemed healthy. Nevertheless, there is considerable evidence that PBM can be used to treat the symptoms of sensitive dentine [32, 33], and hence PBM protocols can be adjusted to consider this.

Additionally, PBM has been proposed for reversal of dental anesthesia. The primary mechanism by which this is achieved is increase in blood flow, allowing clearance of local anesthetic agent. This is especially important in candidates who are at high risk of iatrogenic trauma while under the influence of anesthetic. As opposed to the wavelengths used in this study that produced analgesia, lower wavelengths between 600 and 800 nm are better absorbed by water and hence are utilised to increase blood flow in the area where anesthesia is to be reversed [34]. The impact of wavelength is therefore highly important, and reporting of all parameters is considered prudent for reproducibility of the results [35].

## Conclusions

This study compared four PBM sources for their effectiveness in producing dental analgesia, indicated by an increase in EPT score. The multi-wavelength LED source was the most effective, followed by the 904 nm diode laser. The onset time was 1–2 min for the LED and 904 nm, and 2–5 min for the 808 nm and 660 nm diode lasers. The LED source was also the best tolerated, with greatest number of responders, and produced the least discomfort. Further studies are required to assess what dental procedures may be done with comfort with analgesia produced by PBM.
